# Cancer Stem Cell Quiescence and Plasticity as Major Challenges in Cancer Therapy

**DOI:** 10.1155/2016/1740936

**Published:** 2016-06-21

**Authors:** Wanyin Chen, Jihu Dong, Jacques Haiech, Marie-Claude Kilhoffer, Maria Zeniou

**Affiliations:** Laboratoire d'Innovation Thérapeutique, Université de Strasbourg/CNRS UMR7200, Laboratoire d'Excellence Medalis, Faculté de Pharmacie, 74 route du Rhin, 67401 Illkirch, France

## Abstract

Cells with stem-like properties, tumorigenic potential, and treatment-resistant phenotypes have been identified in many human malignancies. Based on the properties they share with nonneoplastic stem cells or their ability to initiate and propagate tumors* in vivo*, such cells were designated as cancer stem (stem-like) or tumor initiating/propagating cells. Owing to their implication in treatment resistance, cancer stem cells (CSCs) have been the subject of intense investigation in past years. Comprehension of CSCs' intrinsic properties and mechanisms they develop to survive and even enhance their aggressive phenotype within the hostile conditions of the tumor microenvironment has reoriented therapeutic strategies to fight cancer. This report provides selected examples of malignancies in which the presence of CSCs has been evidenced and briefly discusses methods to identify, isolate, and functionally characterize the CSC subpopulation of cancer cells. Relevant biological targets in CSCs, their link to treatment resistance, proposed targeting strategies, and limitations of these approaches are presented. Two major aspects of CSC physiopathology, namely, relative* in vivo* quiescence and plasticity in response to microenvironmental cues or treatment, are highlighted. Implications of these findings in the context of the development of new therapies are discussed.

## 1. Scope of This Review

Many if not most cancers are characterized by the presence of a subpopulation of tumor cells endowed with tumor initiation and propagation ability and a physiopathological state leading to great resistance to conventional therapies. Because it was initially presumed that such cells originated from malignant transformation of normal stem cells and in view of their tumorigenic potential, these cells were designated as cancer stem (or stem-like) cells or tumor initiating/propagating cells. In this review they will be referred to as cancer stem cells (CSCs).

Isolation and subsequent studies of CSCs from different types of tumors pointed to these cells as major components of conventional treatment failure. As a consequence, targeting CSCs is a promising perspective for the development of novel more effective anticancer therapeutic protocols. In this context, great efforts are made to identify and develop new anti-CSC therapies. However, the more we learn about CSCs, the more it becomes obvious that targeting this particular cancer cell subpopulation will be challenging.

Cancer cells endowed with stem cell properties are maintained* in vivo* in a quiescent slow-growing state which preserves them from antiproliferating anticancer drugs. In addition, CSC function is elusive and may be enhanced or modified by environmental cues or treatment. Moreover, these modifications may occur in only a part of these cells leading to CSC heterogeneity within the same tumor. More importantly, normal or cancer cells without stem cell properties may be induced to treatment-resistant CSCs depending on signals from their microenvironment.

This review will describe CSCs' functional characteristics and some methods used for their identification. Relevant biological targets in CSCs will be presented with a focus on quiescence and plasticity, two major aspects of CSCs' physiopathology. Data presented aim to highlight future challenges in CSC targeting and elimination in order to eradicate tumors.

## 2. Malignancies with Hierarchical Organization and CSCs

Evidence for the presence of cancer cells with stem cell properties in human malignancies was provided by Bonnet and Dick in the late nineties. These authors described CD34^+^/CD38^−^ cancer cells able to initiate acute myeloid leukemia in immunocompromised mice. They postulated that these cells originate from oncogenic transformation of hematopoietic stem cells since they presented similarities in cell surface marker expression, proliferation, self-renewal, and differentiation abilities [1]. This discovery is at the basis of the hierarchical or cancer stem cell (CSC) model postulating that tumors are hierarchically organized with CSCs at the apex of this hierarchy. CSCs would be unique among cancer cells through their ability to sustain* in vivo* long-term tumorigenic potential [2]. It is of note that the CSC model does not imply that CSCs arise from oncogenic transformation of normal stem cells since any cell in the hierarchy with proliferative ability could be at the origin of CSCs and thus of tumors [3, 4]. This hierarchical or CSC model was initially opposed to the clonal evolution theory suggesting that all undifferentiated cells within a tumor have equal tumorigenic potential provided by random additional mutations or epigenetic modifications [5]. Experimental data demonstrating that non-CSC populations may acquire CSC functionalities depending on the cell environmental context [6–8] supports the idea that the CSC and clonal evolution models present much more similarities than initially proposed.

Based on surface marker expression patterns, sphere formation ability, side population detection, and* in vivo *tumorigenic potential following serial transplantation [9], cancer stem cells have been subsequently isolated from numerous solid tumors, the first one being breast carcinoma [10, 11]. Additional solid tumors that adhere to the hierarchical or CSC model include, but are not limited to, brain [12–16], pancreatic [17, 18], colon [19–21], head and neck [22], hepatic [23], lung [24, 25], prostate [26], bladder [27], and ovarian malignancies [28, 29], as well as melanoma [30–32] and musculoskeletal sarcomas [33].

## 3. CSC Properties. What Defines a CSC?

**Figure 1:**
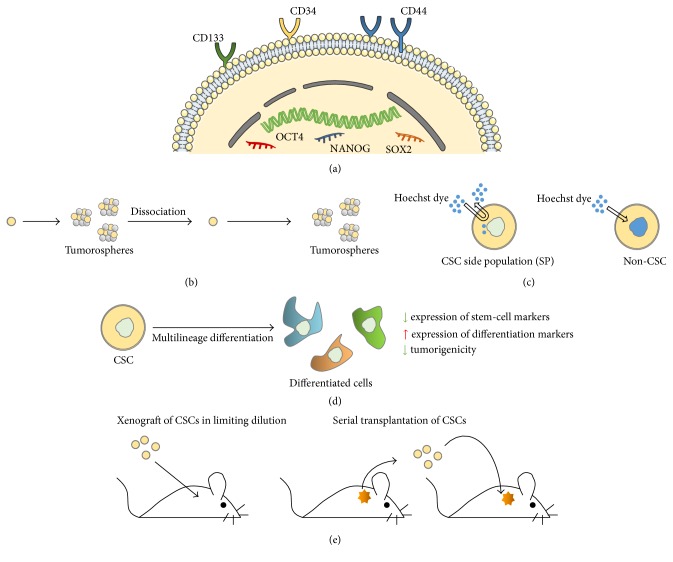
Cancer stem cell (CSC) properties and experimental methods for phenotypic and functional characterization of CSCs. (a) CSCs express cell surface antigens (CD133, CD34, and CD44) and signaling molecules (OCT4, NANOG, and SOX2) linked to a stem-like phenotype. CD: cluster of differentiation; OCT-4: octamer-binding transcription factor 4. (b) CSCs possess clonal properties and may be maintained* in vitro* for long intervals in serum-free medium. Under these conditions, they are able to form clonal tumorospheres. (c) CSCs present increased Hoechst efflux capacity compared to normal cells. Based on this property, they are designated as the side population (SP). (d) Multilineage differentiation (in the presence of serum) is another property of CSCs. Differentiation ability is verified by the decrease in the expression of stem cell markers accompanied by an increase in the expression of differentiation markers. Differentiated cells lose their tumorigenic potential. (e) CSCs at limiting dilutions are able to generate tumors after serial xenografting into immunocompromised mice. These tumors recapitulate the characteristics of the tumor from which CSCs were derived. Figure was constructed in part with objects from Servier Medical Art documents under license from Creative Commons Attribution 3.0 France (http://creativecommons.org/licenses/by/3.0/fr/legalcode).

Cell surface antigen expression profiles have been commonly used for enrichment of CSCs from tumors (Figure 1(a)). In acute myeloid leukemia, CSCs were shown to express CD34 (Cluster of Differentiation 34) and lack expression of CD38 [1, 34]. CD133 (prominin 1) expression was described and used for isolation of CSCs from various solid tumors including glioblastoma [15], prostate [26], colon [20, 21], lung [24], pancreatic [17], and ovarian cancers [35] and melanoma [36]. The CD44 adhesion molecule is another surface marker that has often been associated with CSC phenotypes. CSCs showing expressions of CD44 were described, for example, in breast [10], colorectal [19], pancreatic [18], and ovarian malignancies [29]. Several other surface markers have been used to define CSCs (reviewed in [37–39]). However, surface marker expression profiles may vary during tumor growth* in vivo* and as a function of experimental conditions* in vitro*. In addition, these markers often lack specificity and heterogeneity in marker expression may exist between patients and even within the same tumor [39]. Combining different markers was proposed to improve reliability of this type of approach. Alternatively, new, functional markers, most of which are associated with the intrinsic stem properties of CSCs, have been used. Increased ALDH1 (aldehyde dehydrogenase 1) expression was, for example, used as a marker of CSCs from mammary tumors [11] as well as of CSCs from bladder, lung, colon, esophageal, and head and neck squamous cell carcinomas [38]. Although not restricted to CSCs, expression of components of signaling pathways associated with cell pluripotency was also used to characterize these cells. Proteins whose expression was linked to the stem-like phenotype of CSCs include the OCT-4 (octamer-binding transcription factor 4), SOX2, and NANOG transcription factors (Figure 1(a)) as well as components of the Wnt/*β*-catenin, Notch, and Hedgehog (Hh) signaling modules [40, 41]. Reverse transcription followed by real-time polymerase chain reaction (PCR) is an extremely sensitive method commonly used for CSC marker identification. In this approach, expression levels of the gene(s) of interest need to be normalized against endogenous control genes (housekeeping genes) whose expression should be robust and highly stable in the experimental conditions used. In a recent study, the stability of 15 commonly used housekeeping genes was evaluated in CSC spheroids from musculoskeletal sarcomas and carcinomas and from breast and renal malignancies as well as in the corresponding adherent native tumor cell lines [42]. Housekeeping genes encoding Tata-Binding Protein (TBP), Tyrosine 3-monooxygenase/tryptophan 5-monooxygenase activation protein zeta polypeptide (YWHAZ), peptidylprolyl isomerase A (PPIA), hydroxymethylbilane synthase (HMBS), or GAPDH (glyceraldehyde-3-phosphate dehydrogenase), were shown to be appropriate for comparative expression studies in cells used in this report. In addition, the authors suggested that more than one endogenous control gene should be used for normalization and that different, specific housekeeping genes should be considered for distinct CSCs and/or as a function of experimental conditions [42]. Recent data obtained in our laboratory with human glioblastoma CSCs and control cells further argue in favor of the necessity to validate appropriate housekeeping genes for each experimental setting [43, 44].

Since marker expression is definitely not sufficient to define a specific CSC subpopulation, it is now a consensus that phenotypic characterization must be accompanied by functional validation of CSCs [45, 46]. Thus, in addition to cell surface marker and stem cell marker expression profiling (Figure 1(a)) [9], various methods have been developed* in vitro* and* in vivo* to assess the stem cell properties of cells. Sphere formation assays following limiting dilution of cells are used for* in vitro* evaluation of cells' self-renewal and proliferation abilities (Figure 1(b)). Based on their increased efflux capacity of the Hoechst dye, mediated by overexpression of ATP-binding (ABC) cassette transporters, CSCs are designated as the side population (SP) cells (Figure 1(c)) [47–49]. Differentiation potential is demonstrated by the ability of cells to undergo morphological changes when exposed to serum and by modifications in expression levels of stem cell and differentiation markers. In the differentiated state, cells lose their tumorigenic properties (Figure 1(d)). Finally, the gold standard for CSC validation is the* in vivo* ability of limiting dilutions of cells to recapitulate the heterogeneity and complexity of the initial tumor following serial orthotopic or ectopic transplantation in appropriate animal models (Figure 1(e)) [9, 38, 50]. Master and working cell banking of isolated CSCs, limited numbers of passages, and DNA fingerprinting are recommended to achieve nonderivation of CSCs from initial phenotypic and functional phenotypes* in vitro*. Several CSC properties and associated validation methods are shown in Figure 1.

## 4. Well Established Biological Targets in CSCs Related to Treatment Resistance

### 4.1. Self-Renewal Signaling Molecules

**Figure 2:**
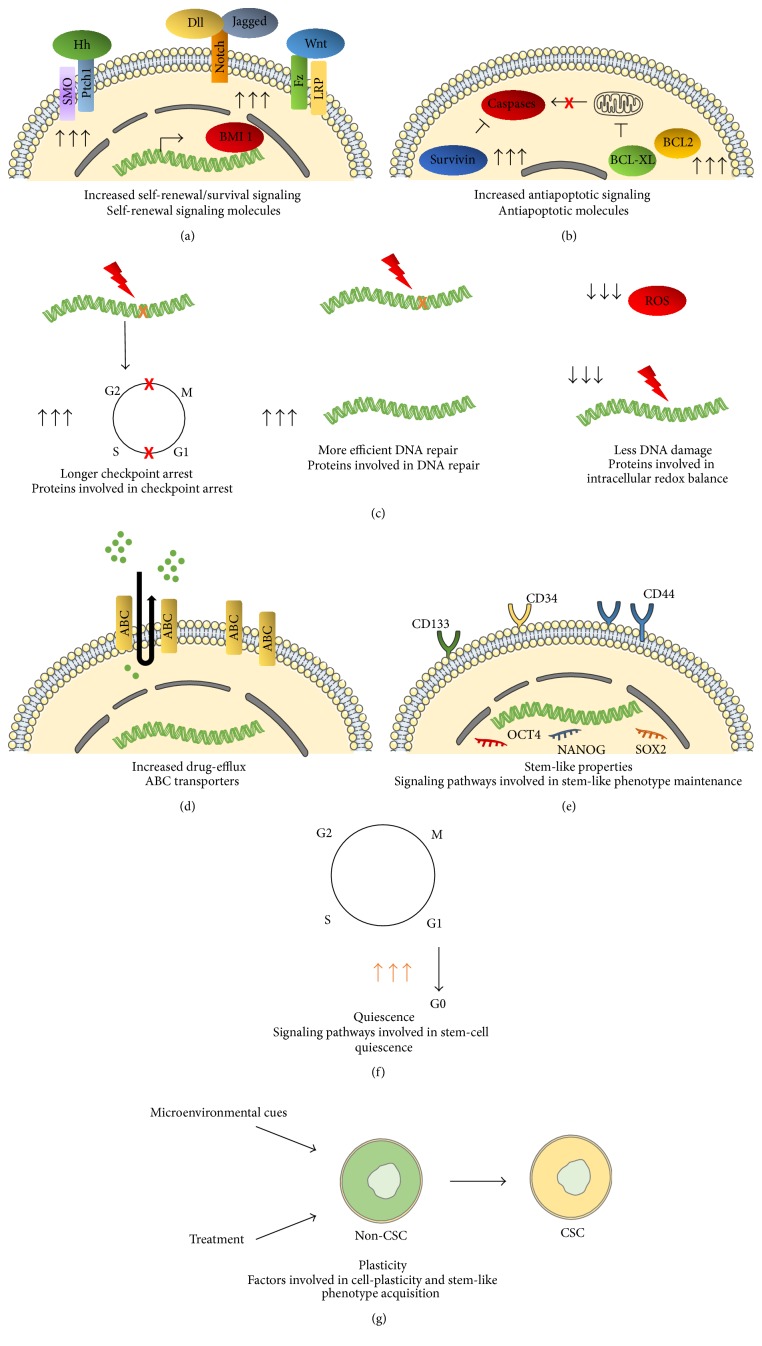
Mechanisms of CSCs' therapy resistance and relevant biological targets. ((a)-(b)) Increased self-renewal and prosurvival signaling have been reported for CSCs. Molecules involved in self-renewal/survival as well as antiapoptotic proteins overexpressed in CSCs are potentially interesting targets for therapies seeking CSC elimination. Hh: Hedgehog; SMO: Smoothened; Ptch1: Patched; Dll: Delta-like ligand; Wnt: wingless integration site; Fz: Frizzled; LRP: low-density lipoprotein receptor-related protein; BMI1: polycomb ring finger; BCL-XL: B-cell lymphoma extra-large; BCL2: B-cell lymphoma 2. (c) CSCs respond with higher efficacy to DNA damage* via* checkpoint arrest for longer time intervals and enhanced DNA repair. Moreover, reduced levels of ROS have been reported in CSCs leading to protection of the CSC genome from DNA damage. Proteins involved in checkpoint arrest, DNA repair, and intracellular redox balance are relevant biological targets in CSCs. (d) Increased expression/function of ABC transporters in CSCs underlies more efficient drug efflux from these cells. ABC transporters are thus interesting therapeutic targets in CSCs. (e) Tumor initiation and propagation properties of CSCs involve their stem-like phenotype. Signaling modules involved in the maintenance of this state are relevant targets for CSC elimination. CD: cluster of differentiation; OCT-4: octamer-binding transcription factor 4. (f) Quiescent CSCs have been evidenced in many human malignancies and are major determinants of CSCs' resistance to current treatments. Neutralization of the CSC quiescent phenotype is a promising approach for new anticancer protocols. (g) Induced CSC-like phenotypes may be obtained by the action of signals from the tumor microenvironment and/or as a result of therapy. Cell plasticity observed in human malignancies must be taken into account when developing new anticancer therapies. Figure was constructed in part with objects from Servier Medical Art documents under license from Creative Commons Attribution 3.0 France (http://creativecommons.org/licenses/by/3.0/fr/legalcode).

One of CSC's major features is their long-term self-renewal ability both* in vitro* and* in vivo*. Several pathways well known for their implication in embryonic development and differentiation and controlling stem cell self-renewal are preferentially activated in CSCs. These include the Wnt/*β*-catenin, Notch, Hedgehog (Hh), and BMI1 pathways (Figure 2(a)) [51]. In addition, several studies suggest that EGF (epidermal growth factor), PI3K (phosphatidylinositol-3-phosphate kinase), MAPK (mitogen-activated protein kinase), and NF-*κ*B- (nuclear factor kappa B-) mediated signaling is also involved in CSC self-renewal through cross-talk with the aforementioned pathways [52]. As a consequence, several strategies have been developed to target these pathways and a number of their inhibitors and/or regulators are under clinical investigation and some are already in clinical use [51, 53, 54]. However, various resistance mechanisms to these experimental drugs have been described. For example, resistance to inhibitors of Smoothened, a key molecule in the Hedgehog signaling pathway, has been attributed to activation of Smoothened downstream signaling partners by other signaling pathways [55]. Moreover, Smoothened mutations causing disruption of the inhibitor binding site were also described [56]. In addition to resistance mechanisms, some examples of absence of clinical efficacy have been reported [54, 57]. These concerned trials without previous patient stratification for mutations in the targeted pathways. Absence of efficacy was also attributed to the fact that the CSC phenotype is not only the result of dysregulation of a single pathway but also the outcome of cross-talk between multiple signals. Thus, targeting one single pathway may not be sufficient [54].

### 4.2. Antiapoptotic Pathways

Overexpression of antiapoptotic molecules is another feature of CSCs (Figure 2(b)). For example, overexpression of genes encoding BCL2 and BCL-XL, which act as negative regulators of mitochondrial membrane permeabilization and cytochrome C release [58], was reported in CSCs from high grade astrocytomas. In addition, higher levels of survivin, belonging to the IAP (inhibitors of apoptosis) family members and low mRNA levels of caspase 8 associated with TRAIL (tumor necrosis factor- (TNF-) related apoptosis-inducing ligand) resistance were described in other brain tumor-derived CSCs [59, 60]. Moreover, CSCs from glioblastoma, the most common and aggressive brain malignancy [61, 62], were less sensitive to BCL2 small molecule inhibitors compared to cancer cells without stem cell properties from the same tumor [63]. Interestingly, specific inhibition of antiapoptotic pathways was shown to reduce chemo- and radioresistance of glioblastoma CSCs and to specifically eliminate breast cancer stem cell activity [64, 65]. However, presumably because of the coexistence of multiple antiapoptotic mechanisms, clinical trials based on the use of death receptor agonists were not conclusive. Association of these compounds with other antiapoptotic molecules such as BCL2 antagonists may be more relevant for efficient tumor targeting and elimination [53].

### 4.3. Resistance to DNA Damage and Proteins Involved in DNA Repair

Lesions induced by radiotherapy and chemotherapy with DNA damaging agents need to be repaired by the DNA damage response (DDR) pathways to allow cancer cells' survival following treatment. DDR mechanisms include both arrest of cells at specific checkpoints of the cell cycle and recruitment of the DNA repair machinery leading to elimination of lesions. Depending on the type of lesion, distinct sets of DNA repair proteins are involved [66].

In a pioneer study, Bao and coll. showed that CD133^+^ glioblastoma CSCs contribute to radioresistance through preferential activation of the DNA damage checkpoint response (Figure 2(c)) involving the ataxia telangiectasia mutated (ATM) protein kinase and checkpoint kinase (Chk) 2 (Chk2). Interestingly, the radioresistance of these cells was reversed following specific inhibition of Chk1 and Chk2 [67]. Chk1 inhibitors were also able to sensitize pancreatic adenocarcinoma CSCs to gemcitabine, a cytidine analog used for the treatment of several malignancies including those of the pancreas [68].

In addition to activation of checkpoint responses, enhanced DNA repair mechanisms were reported for some types of CSCs (Figure 2(c)). Preferential activation of the DNA double strand break (DSB) repair response involving the polycomb group protein BMI1 and the ATM protein kinase was observed in cancerous neural stem cells [69]. BMI1 deficiency resulted in increased sensitivity to radiation in both glioblastoma and head and neck squamous cell cancer-derived CSCs [69, 70]. Preferential expression of other DNA repair-associated genes such as those encoding Methyl Guanine Methyl Transferase (MGMT) and BRCA1-related DNA repair proteins was reported in CSCs from glioblastoma and pancreatic tumors, respectively [71, 72]. Moreover, enhanced Non-Homologous End Joining (NHEJ) activity and a more-rapid DNA repair were reported in CSCs from mammary tumors [73].

Finally, CSCs' chemo- or radioresistance was also associated with lower levels of reactive oxygen species (ROS) either because of lower ROS production rates or because of the presence of more efficient ROS scavenging systems in these cells involving multiple signaling pathways [74]. Interfering with CSCs' intracellular redox balance is thus an interesting approach for CSC elimination (Figure 2(c)) [75, 76].

### 4.4. Proteins Involved in Drug Efflux

One of the characteristics of CSCs is their increased efflux capacity of the Hoechst dye defining them as the side population (SP) cells (Figure 1(c)) [47–49]. This property has been used and is still applied for isolation of CSCs from a variety of tumors [77–79]. Increased expression of proteins belonging to the ATP-binding (ABC) cassette transporter family is at the basis of this CSC property (Figure 2(d)) [80]. Among ABC transporters, P-glycoprotein (also known as multidrug resistance protein 1 (MDR1) or ABCB1), multidrug resistance associated proteins 1 and 2 (MRP1 or ABCC1 and MRP2 or ABCC2), and breast cancer resistance protein (BCRP or ABCG2) are main actors of the multidrug resistance (MDR) phenotype which was also associated with CSC physiopathology [80]. Overexpression of ABC family members was described in glioblastoma, lung cancer, osteosarcoma, prostate and ovarian cancer, and nasopharyngeal carcinoma [80]. Three generations of ABC transporter blockers have been used. A fourth one based on natural compounds is under development [81, 82]. Inhibitors of the third generation which are less toxic and more specific, namely, the ones targeting BCRP, are under investigation in clinical trials whereas others are already used in clinic, for example, in colon carcinoma [53, 80].

### 4.5. Stem-Like Properties

Tumorigenic properties of CSCs and treatment resistance are closely linked to their undifferentiated phenotype and stem-like characteristics (Figure 2(e)). It was thus proposed that inducing CSC differentiation would be an efficient way to increase therapy efficacy. Differentiation therapy with various agents including all-trans retinoic acid and vitamin D3 has been proposed in the context of hematopoietic malignancies [83]. SAHA (suberoylanilide hydroxamic acid), a histone deacetylase (HDAC) inhibitor, was used to induce differentiation of various cancer cell types including those of breast and endometrial carcinomas [83]. CSCs isolated from glioblastoma and treated* in vitro* with bone morphogenetic protein 4 (BMP4) expressed higher levels of differentiation markers and lost their ability to generate glioblastoma-like lesions in xenografted mouse brains. These effects were dependent on Smad signaling [84]. However, some glioblastoma CSCs were subsequently shown to resist BMP4-induced differentiation because of epigenetic silencing of BMP receptors in these cells [85]. In a recent communication, Balasubramaniyan et al. reported that glioblastoma CSCs retained their self-renewal and tumorigenic properties despite the induction of proneural differentiation factors by exposure to serum. Moreover, aberrant differentiation towards a mesenchymal phenotype was observed [86].

## 5. Emerging Biological Targets and Treatment Resistance of CSCs

### 5.1. Cell Quiescence

Cell quiescence may be defined as a reversible G0 phase from which cells may escape to reenter the cell cycle in response to physiological cell stimuli. It was suggested that cell quiescence is not just a passive state but rather a condition actively maintained and regulated by signaling pathways allowing rapid activation of quiescent cells and reentry in the cell cycle [87].

Signaling molecules participating in the regulation of stem cell quiescence include tumor suppressors p53 and RB (retinoblastoma protein), cyclin-dependent protein kinase inhibitors, namely, p21, p27, and p57, Notch-related pathways, and a number of miRNAs (micro-RNAs) [87]. Several transcription factors including FoxOs (Forkhead Box O) and NFI (Nuclear Factor 1) protein member NFIX have also been involved in gene expression regulation in quiescent cells [88, 89].

Specific strategies allowing long-term survival through adaptive responses to environmental stress were reported for quiescent CSCs. For example, FoxO transcription factors,* via* PI3K-Akt-dependent pathways and regulation of ROS levels, were shown to participate in such adaptive mechanisms. Adaptive metabolic responses and mechanisms favoring maintenance of genomic integrity were also reported for these cells [87, 90, 91].

In cancer biology, tumor dormancy designates a frequent clinical phenomenon in which disseminated tumor cells are maintained in a nonproliferating quiescent state for long time intervals. This phenomenon may occur at early stages of the disease or following therapeutic intervention. Awakening of these dormant cells leads to tumor progression and relapse which may occur after very long periods [91, 92].

In addition to disseminated dormant tumor cells, CSCs with quiescent phenotypes also exist within tumors as suggested by* in vitro* and* in vivo* data. In 2009, based on the label retention properties of cells, Dembinski and Krauss identified a subpopulation of slow cycling cells in pancreas adenocarcinoma cell lines. Partial overlap between this subpopulation and stem cell marker expression was observed for some cancer cells. Interestingly, these cells survived following chemotherapy and exhibited increased tumorigenic and invasive potentials [93]. A label retention strategy was also used to identify, purify, and establish transcriptional signatures of quiescent normal mammary stem cells from cultured mammospheres. Transcriptional signatures of these cells allowed prospective identification of slowly dividing CSCs in breast tumors and highlighted the heterogeneity of such malignancies with respect to their CSC content [94]. In liver cancer, the cell surface marker CD13 was identified as a functional hallmark of potentially dormant CSCs. CD13^+^ cells retain dyes for long intervals, contain low levels of ROS, participate in chemoresistance, and present high tumorigenic potential in immunocompromised mice. In* in vivo *liver tumor models, combination of CD13 inhibition and 5 fluorouracil (5-FU) damaging cells in the S phase of the cell cycle led to tumor volume reduction in a more effective way compared to either treatment alone [95]. JARID1B which is a histone 3 demethylase involved in transcriptional repression of Notch ligands was identified as a marker of temporarily distinct slow cycling melanoma cells. Targeting the slow cycling phenotype of these cells through JARID1B knockdown inhibited continuous growth and metastatic progression of melanomas in animal models [96]. Relatively quiescent CSCs were also isolated from ovarian cancer patient specimens [97] as well as from the colo205 human colon adenocarcinoma cell line [98]. Label retaining glioblastoma CSCs generating tumors which present all the pathological features of the primary disease were first described by Deleyrolle et al. [99]. Endogenous glioblastoma CSCs, expressing a transgene that labels quiescent adult neural stem cells of the subventricular zone and staining negative for proliferation marker Ki-67 expression, were subsequently identified by Chen et al. in a genetically engineered mouse model of glioma. Following treatment with temozolomide (TMZ), one of the standards of care for glioblastoma together with surgery and radiotherapy, this cancer cell subpopulation was able to drive tumor regrowth through the production of highly dividing cells. Interestingly, ablation of this particular cancer cell subpopulation hindered tumor development [100]. In another study, Patel et al. used RNA seq-based single cell transcriptomic analysis to demonstrate that glioblastomas are highly heterogeneous tumors harboring variable proportions of cells expressing markers that have previously been associated with quiescence. These cells are also characterized by the presence of a stemness signature which is attributed to glioblastoma CSCs [101]. Finally, HIF1*α*- (hypoxia-inducible factor 1 alpha-) positive quiescent glioblastoma cells with stem properties were localized by immunocytochemical-based methods in perinecrotic niches in glioblastoma patient specimens. Suppressed phosphorylation of serine 2 in the CTD (C terminal domain) of RNA polymerase II, previously observed in various types of quiescent noncancerous stem cells, was used as an indicator of quiescence in their report [102].

**Figure 3:**
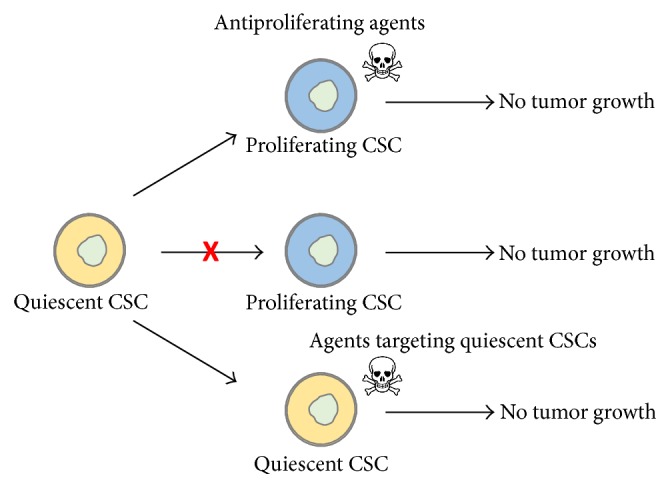
Strategies proposed for neutralization of the CSC quiescent phenotype involved in treatment resistance. Three strategies have been proposed to neutralize the quiescent CSC-state. Induction of CSC entry into the cell cycle would sensitize cells to antiproliferating agents. Blocking quiescent CSCs in G0 was proposed as an alternative for preventing new tumor growth. Targeting CSCs in the quiescent state was also proposed for the elimination of this particular CSC subpopulation. CSC: cancer stem cell. Figure was constructed in part with objects from Servier Medical Art documents under license from Creative Commons Attribution 3.0 France (http://creativecommons.org/licenses/by/3.0/fr/legalcode).

The quiescent state of CSCs protects these cells from antiproliferating agents and is thus an important factor of CSC-related resistance to conventional therapy (Figure 2(f)). Three major strategies have been reported for targeting this particular slow cycling CSC subpopulation (Figure 3). The first one consists in forcing CSCs to reenter the cell cycle and was designated as the “locked-out” situation. This was suggested to be of benefit for cancer treatment since a majority of chemotherapeutic agents including mitotic inhibitors, antimetabolite drugs, and topoisomerase inhibitors may only exhibit cancer cell cytotoxicity on proliferating cells. For example, ablation of the F-box protein Fbxw7 leads to a decrease in ubiquitin-dependent degradation of c-Myc, Notch, and cyclin E and reentry in the cell cycle and increases the sensitivity of Phi + leukemia CSCs to imatinib [103]. Leukemia CSCs were also sensitized to cell cycle dependent chemotherapy after treatment with mitogens (GCSF) [104]. However, the “locked-out” approach might be risky in case all awakened cancer cells are not efficiently eliminated by available antiproliferating agents since this would lead to disease progression. Moreover, exit from dormancy of heterogeneous populations of cancer cells may increase the genetic and epigenetic complexity of the tumor and allow more efficient resistance to treatment [92]. To overcome these limitations, some authors have proposed alternative targeting strategies. One of them is the “locked-in” strategy in which pharmacological maintenance of CSCs in the G0 phase aims to prevent further tumor growth, relapse, and/or metastasis throughout the lifetime of a patient. Eradicating CSCs while they are dormant is another alternative to dormant cancer cell awakening.

Deeper understanding of signaling pathways and factors involved in cell quiescence is a prerequisite to the success of those latter strategies. For example, reduced PI3K-AKt signaling was associated with dormant phenotypes. In addition, inhibition of mitogenic signals was shown to trigger quiescence. Combining cell survival blockers, that is, ABT-737, a BCL2, and BCL-XL inhibitor to EGFR (Epidermal Growth Factor Receptor) inhibition by erlotinib, was able to lead to elimination of erlotinib-induced quiescent cells in non-small-cell lung cancer xenografts. Alternatively, the quiescent state may be actively induced by specific kinases including DYRK1B (dual specificity tyrosine phosphorylation-regulated kinase 1B). This kinase was shown to block proteins involved in the G0/G1/S transition. DYRK1A, a DYRK1B related kinase, can also induce quiescence together with coordinating survival* via* an antioxidant response. Inhibition of DYRK1A leads to cytotoxicity towards quiescent pancreatic cancer cells while preserving normal quiescent cells. The underlying mechanisms are not known [92]. The p38 MAPK (Mitogen-Activated Protein Kinase) along with TGF*β* (Transforming Growth Factor beta)/BMP (Bone Morphogenetic Protein) signaling were also involved in the maintenance and/or induction of the quiescent state [105–107]. The DNA methylation inhibitor 5-azacytidine was shown to cause a decrease in expression of genes involved in exit from the G0 phase and entry in G1 in primary cells and in leukemia and breast cancer cell lines and upregulation of genes involved in a p38-related dormancy signature [105]. The same authors reported that combinations of 5-azacytidine and all-trans retinoic acid induce a stable quiescent state which may be maintained for a long period of time [92]. We have recently developed an* in vitro* model of reversibly quiescent glioblastoma CSCs based on the maintenance of patient derived CSCs without medium renewal for several days. These cells were shown to present decreased EdU (5-ethynyl-2′-deoxyuridine) incorporation rates and very low levels of Ki-67 expression. No significant increase in the expression of apoptotic markers was observed in these conditions. We additionally showed that quiescent glioblastoma CSCs showed similar expression of surface markers and comparable* in vitro* sphere forming and differentiation abilities, when returned to proliferation or differentiation-promoting culture conditions, as their proliferating counterparts. Moreover,* in vivo* engraftment capacity was maintained. Screening of the Prestwick Chemical library, mainly composed of FDA-approved drugs currently used in various therapeutic domains, on proliferating and quiescent glioblastoma CSCs, led to the identification of the stimulant laxative bisacodyl as a potent and specific inhibitor of quiescent glioblastoma CSC survival, with an IC50 value around 1 *μ*M. Bisacodyl was ineffective on proliferating CSCs from the same patient, as well as on normal fetal neural stem cells and primary astrocytes [44]. To our knowledge, no other small molecules with similar activity profiles have been reported so far. The molecular mechanisms underlying bisacodyl's activity on quiescent glioblastoma CSCs are currently under investigation in our laboratory.

### 5.2. CSC Plasticity

Initially, CSCs were considered as a static well-defined subpopulation of cancer cells with invariable functional characteristics distinguishing them from cells of the tumor mass. Nowadays, the CSC phenotype is considered as a transient state that any cell may acquire depending on cues provided by its microenvironment (Figure 2(g)) [108]. Epigenetic modifications are a major source of this kind of cell plasticity. Genomic alterations and selection of mutant cells may also participate in this phenomenon [109, 110].

Cell plasticity with acquisition of stem-like properties was described in several cancers. In melanoma, it was shown that many phenotypically distinct types of cancer cells with respect to surface marker expression were able to form tumors that recapitulate the characteristics of the original malignancy. This suggested that tumorigenic cells may undergo reversible phenotypical changes* in vivo* [111]. In addition, Roesch and colleges described a slow cycling melanoma CSC subpopulation whose existence within the tumor bulk was regulated over time as evidenced by marker expression modifications [96]. In another study, genetically engineered transformed mammary epithelial cells were shown to spontaneously generate cancer stem-like cells both* in vitro* and* in vivo* [6]. The same authors subsequently showed that switching of human basal breast cancer cells from a non-CSC to a CSC-state may be achieved through mesenchymal phenotype-inducing signals [7]. More recently, reprogramming of the tumor propagating potential of differentiated glioblastoma cells was achieved through expression of a set of transcription factors involved in neuronal development, namely, POU3F2, SOX2, SALL2, and OLIG2 [112]. Induced CSCs were also obtained from colon cancer cells through introduction of three factors, OCT3/4, SOX2, and KLF4 [113].

**Figure 4:**
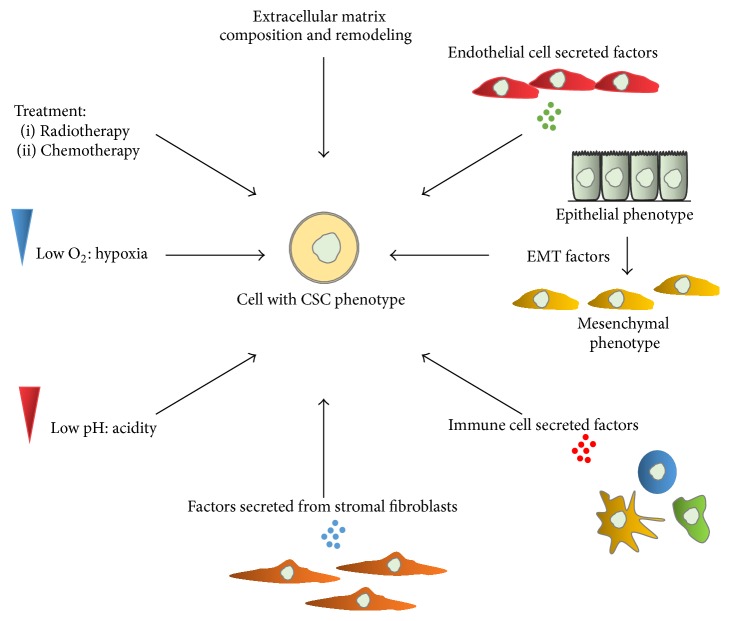
Mechanisms inducing CSC phenotypes. The CSC phenotype of cancer cells is influenced by cues related to the tumor microenvironment. These include remodeling of the extracellular matrix and signaling through factors secreted by endothelial, immune system cells and stromal fibroblasts. Signaling related to EMT (epithelial to mesenchymal transition) may also induce a CSC phenotype. Low oxygen (hypoxia) and acidic conditions nearby the CSC niche may induce and/or enhance the CSC phenotype of cells. Radiotherapy and chemotherapy have been shown to induce dedifferentiation of cancer cells and acquisition of a CSC phenotype. Figure was constructed in part with objects from Servier Medical Art documents under license from Creative Commons Attribution 3.0 France (http://creativecommons.org/licenses/by/3.0/fr/legalcode).

Cancer cell plasticity with respect to the acquisition or loss of stem-like properties may be induced by either microenvironmental-/niche-derived signaling cues and/or as a result of antitumor therapeutic intervention (Figure 4). Tumors may be considered as organ-like structures in which cancer cell function is supported/regulated by matrix remodeling, blood vessel development, cancer associated fibroblast function, and recruitment of immune cells [114, 115]. Each of these factors may contribute to the functional properties of cancer cells and affect cell response to chemo- and radiotherapy by protecting cells from these agents [108]. It is important to note that vascular supply, access to growth factors, structural support, and interactions with immune cells vary within a single tumor. In other words, all cancer cells do not share the same microenvironmental conditions or the same niche [114]. Some authors suggested that within tumors, CSCs reside in particular niches whose function is to preserve their functional properties and plasticity and facilitate their metastatic potential [116]. In addition, it was proposed that CSC niches may be modified as a function of tumor stage or nature (initial or metastatic) [117].

Microenvironmental signals that can regulate cancer stem cell fate and metastatic potential include reorganizations of the extracellular matrix, autocrine and paracrine factors, low oxygen (hypoxia), and/or nutrient supply and signals derived from immune cells.

Extracellular matrix composition and remodeling have been associated with many aspects of cancer physiopathology as well as in the regulation of stem cell fate [118–120]. In a recent study, extracellular matrix small leucine-rich proteoglycans (SLRPs) decorin and lumican were shown to be expressed at higher levels in glioblastoma and neuroblastoma cancer cells induced* in vitro* to a more stem-like phenotype. Resulting cells forming neurospheres had a slow cycling phenotype and were more resistant to treatment [121].

A role of tumor microvascular endothelial cell-secreted factors on the induction of a stem cell phenotype in differentiated glioblastoma cells was also established. The authors showed that basic fibroblast growth factor (bFGF) secreted by endothelial cells induces increased stem cell marker expression and sphere forming ability of differentiated glioblastoma cells [122].

Epithelial to mesenchymal transition (EMT) is a process initially described in embryogenesis. During this process, differentiated polarized epithelial cells acquire a mesenchymal phenotype and a more motile and invasive behavior [123, 124]. EMT is induced by environmental cues including TGF*β* and receptor tyrosine kinase ligands and is accompanied by complex gene expression modifications [123]. In the context of cancer, EMT was initially described as a mechanism presumably providing cancer cells with invasive and metastatic properties. More recently, similarities between EMT-induced properties and CSC functional characteristics were highlighted and EMT following tumor microenvironmental cues was proposed to result in acquisition of CSC-like phenotypes by cancer cells [123, 125]. For example, it was shown that transformed human mammary epithelial cells acquire CSC properties after undergoing EMT [126, 127]. EMT in this case may be triggered by expression of particular transcription factors, cytokines including TGF*β*, or following an immune response. In addition, in some cases, a stem-like phenotype has been reported for metastatic cancer cells which have presumably undergone EMT [123, 125]. Moreover, exposure of lung cancer cells to TGF*β*1 resulted in the switching of some cells to a stem-like phenotype [128].

Immune cells of the tumor microenvironment were also shown to secrete factors that are able to interfere with the stem cell properties of cancer cells. IL- (interleukin-) 22 secreted by T cells is able to modulate STAT3 signaling in cancer cells and expression of stem cell-associated genes like those encoding NANOG, SOX2, and POU5F1. This leads to increased tumorigenic potential of colorectal cancer cells [129]. Proinflammatory factors such as tumor necrosis factor alpha (TNF*α*) and IL-6, secreted by immune cells in the tumor microenvironment, are able to modify the differentiation state of cancer cells through upregulation of the expression of mesenchymal phenotype-associated genes. This type of stem-like phenotype regulation was described for melanomas and breast and lung cancers. In colon cancer, NF-*κ*B and Wnt activation was linked to dedifferentiation of cells lacking stem-like properties [108] whereas in breast cancer, T cells were shown to promote EMT and acquisition of CSC functions [130].

Interactions between other types of stromal cells and cancer cells were reported in various malignancies including pancreatic, breast, and colon cancer. In pancreatic cancer models, factors secreted from stellate cells (myofibroblasts) induce expression of stem-like or mesenchymal-like fate associated genes and favor CSC phenotypes [131, 132]. In colon cancer, hepatocyte growth factor secreted by myofibroblasts was shown to restore a CSC-like phenotype both* in vitro* and* in vivo *through regulation of the Wnt pathway [133, 134].

One of the hallmarks of solid tumors is the presence of hypoxic regions containing reduced oxygen levels (<2%). Hypoxic zones result from high oxygen demand of cancer cells and low oxygen supply due to irregularities in tumor vascularization or distance from supporting blood vessels [135]. Hypoxia-inducible factors (HIFs) are a family of transcription factors functioning as heterodimers in which one of several *α* subunits (HIF1*α*, HIF2*α*, and HIF3*α*) is associated with a *β* subunit (HIF1*β*). HIF proteins act as major sensors of low oxygen levels. Stabilization of HIF proteins leads to the regulation of the expression of numerous genes involved in pH homeostasis, epigenetic regulation, extracellular matrix remodeling, proliferation, migration, survival, angiogenesis, and cell metabolism regulation by increasing glycolysis and decreasing mitochondrial function [136–138]. Hypoxic conditions were linked to promotion of stem-like properties as well as to EMT [138–140]. Increased expression of stem cell markers was observed in cancer cell lines from prostate, brain, kidney, cervix, lung, colon, liver, and breast tumors subjected to hypoxic conditions. These events were linked to HIF protein stabilization and function [141]. In addition, HIF proteins, and more particularly HIF2*α*, were shown to regulate glioblastoma stem cell properties including sphere formation and tumorigenic potential [142, 143]. Several examples of hypoxia-induced promotion of a dedifferentiated phenotype were reported in breast carcinomas [144, 145]. Moreover, high numbers of tumorigenic cells were localized in hypoxic regions of neuroblastomas [146] and upregulation of CD133, a surface marker linked to CSCs, was reported in medulloblastoma cells under hypoxia [147].

Hypoxia was also linked to the induction of EMT via HIF-dependent or independent mechanisms [148–150]. Increased expression of EMT- and stem cell-related markers was observed in gastric cancer cells cultivated in hypoxic conditions. These cells had higher proliferation and migration rates and were more invasive. Increased ability to form colonies in soft agar was also reported for these cells [151]. In melanoma cells, hypoxic conditions lead to HIF-dependent Snail1 overexpression, decreased E-cadherin levels, and acquisition of melanoma CSC features [152].

One of the functional consequences of low oxygen availability in some regions of a tumor and HIF stabilization is the induction of glycolytic enzymes and a shift from oxidative phosphorylation to glycolysis for energy production. This leads to increased production of metabolic acids such as lactic acid and acidification of the cells' extracellular environment [153]. Acidic stress was also shown to promote CSC-like phenotypes. When CSCs from glioblastoma or CSC-depleted cultures of glioblastoma cells were exposed to low pH, an increase in the expression of CSC markers including OLIG2, OCT4, and NANOG was observed. In addition, cells acquired greater neurosphere formation capacity* in vitro* as well as increased tumorigenicity* in vivo* [154].

Cancer cell plasticity and generation of CSCs from non-CSC-like cancer cells have been frequently reported as a consequence of treatment. Ionizing radiation-reprogrammed breast cancer cells lacking stem cell properties into induced breast CSCs expressing the same markers as their nonirradiated counterparts and possessing increased mammosphere-formation ability and tumorigenic potential [155]. In head and neck squamous cell carcinomas, cisplatin was shown to promote survival and self-renewal of CSCs* in vitro *through increased expression of BMI1 [156]. Melanoma treatment with cisplatin and vemurafenib led to the enrichment of slow cycling cells which are able to sustain long-term tumor growth [157]. Colon cancer cells obtained following 5-FU (5-fluorouracil) treatment present mesenchymal stem-like properties, express stemness markers, and possess spherogenic potential [158]. Finally, several reports have linked chemotherapy to transition into a stem-like phenotype associated with EMT. Some of these reports have been reviewed in [110, 125].

### 5.3. CSC Metastatic Potential

To form metastasis, primary tumor cells need to escape the physical barriers at the primary tumor site, enter the vascular system, infiltrate distant organs, survive, and proliferate at these secondary sites [159]. Because of all these bottlenecks, the metastatic process is rather inefficient and not all primary tumor cells possess metastatic potential. Metastatic potential of tumor cells is dependent both on their origin or their level of differentiation and on the occurrence of genetic and epigenetic changes linked to gene expression modifications that may act as cell fate determinants [160]. For example, in breast cancer, genes and signals involved in metastasis initiation and progression have been described. These include the* TWIST1* gene implicated in EMT, matrix metalloproteinase encoding genes involved in extracellular matrix degradation, genes playing a role in extravasation, in activation of prosurvival and self-renewal signaling pathways, and in initiation of tumor growth at the secondary site [161]. Some metastatic cues may act in a tissue-specific manner, thus directing primary tumor cell initiation of metastatic lesions to specific target organs. The ones driving organ-specific breast cancer metastasis have been reviewed in [161]. Because of their increased prosurvival and self-renewal ability and their tumor initiation properties, CSCs are attractive candidates as tumor cells participating in the metastatic process. Moreover, CSCs have been extensively linked to EMT [123, 125] and subsets of circulating tumor cells with metastatic initiation ability were shown to express high levels of stem cell markers [162–164]. Recently, human metastatic breast cancer cells were shown to possess a stem-like gene expression signature [165]. In addition, loss of differentiation-inducing factors or acquisition of stem cell signaling was related to the development of metastases [160] and CSCs were shown to participate in metastatic colonization by regulating components of their niche at secondary target organs [166]. Metastatic relapse is a major cause of cancer treatment failure. The lack of efficacy of current treatments against metastatic disease was attributed to the presence of genetic alterations in metastatic cells that differ from the ones that are present in primary tumor cells, to the clinical dormancy phenotype of metastatic cells before reamplification at the secondary site as well as to drug resistance induced by treatment. The involvement of treatment-resistant CSCs in this process is also contributing to this phenomenon [160].

## 6. Concluding Remarks

In the past years CSCs have been a topic of intense investigation. As a result, the literature referring to this particular cancer cell subpopulation is very rich and sometimes contradictory. Nowadays, researchers in the field agree that CSCs represent a population of cancer cells with specific properties which definitely distinguish them from cells of the tumor bulk. CSCs are thought to possess or to be able to acquire properties allowing them to resist conventional treatments with great efficacy. Several aspects underlying CSCs' resistance to treatment have been discussed in this review. Because of their resistant phenotype, CSCs have a major implication in tumor relapse following treatment. This has been established for numerous types of malignancies. Thus, new therapies need to target both CSCs and more differentiated cancer cells to be efficient. Intense fundamental and clinical research is developing in this field.

The increasing amount of knowledge concerning signaling pathways and cell mechanisms used by CSCs to sustain their physiopathological functions and induce their resistance to treatment is put to profit for the identification of novel pertinent CSC-targeting strategies. Efforts to target CSCs are however complicated by the probable presence,* in vivo*, of CSCs with a slow-growing status. Such cells will greatly resist antiproliferating molecules disclosed through screening approaches on proliferating CSCs maintained and studied* in vitro*. In the future, development of experimental models of quiescent CSCs will be a prerequisite to understanding specific characteristics of these cells and identifying potentially successful strategies to specifically eliminate the slow-growing CSC subpopulation.

Another critical point is the elusive nature of CSCs. Within a tumor, cells may acquire, enhance, or lose CSC functionalities depending on their microenvironment and challenging by treatment. This implies that any cell within a tumor may become resistant to treatment and that heterogeneity in CSC content and nature may occur within the same tumor or between tumors in distinct patients. CSCs are thus moving targets and their elusive nature needs to be taken into account in future anticancer therapy developments. Combinations of treatments and continuous adaptability to patients' response may be part of the answer.
